# Anthropometric and Biochemical Predictors for Hypertension in a Cross-Sectional Study in Zanzibar, Tanzania

**DOI:** 10.3389/fpubh.2019.00338

**Published:** 2019-11-21

**Authors:** Lara Kim Brackmann, Christoph Buck, Maria Adam Nyangasa, Soerge Kelm, Mohammed Sheikh, Antje Hebestreit

**Affiliations:** ^1^Leibniz Institute for Prevention Research and Epidemiology - BIPS, Department of Epidemiological Methods and Etiological Research, Bremen, Germany; ^2^Leibniz Institute for Prevention Research and Epidemiology - BIPS, Department of Biometry and Data Mangement, Bremen, Germany; ^3^Center for Biomolecular Interactions Bremen, Faculty for Biology and Chemistry, University Bremen, Bremen, Germany; ^4^Environmental Analytical Chemistry and Eco-toxicology Lab, State University of Zanzibar, Zanzibar, Tanzania

**Keywords:** blood pressure, body fat, body mass index, hypertension, salt, sub-Sahara Africa, waist circumference

## Abstract

**Background:** Aim of this study was to describe the proportion of hypertension among Zanzibari of different age-groups and to detect possible correlates of this non-communicable disease.

**Methods:** In 2013 a cross-sectional survey was conducted in Unguja Island, Zanzibar. A total of 235 randomly selected households, including 1,229 (2 to 95 years) eligible study participants, were examined. Association between objectively assessed obesity markers, salt intake and hypertension were investigated. Estimates of 24 h sodium and potassium excretion from a single morning spot urine specimen were calculated and used as surrogate for salt intake. The association between overweight/obesity and hypertension in different age-groups was assessed in multilevel logistic regression models. Further associations between salt intake and hypertension were analyzed.

**Results:** Measures of systolic and diastolic blood pressure as well as proportion of overweight/obesity and hypertension both increased with age. Overweight and obesity were significantly associated with hypertension in adults. Moreover, thinness seems to be associated with hypertension as well. We observed a significantly reduced chance of hypertension for higher urinary sodium-to-potassium compared to a lower ratio in children.

**Conclusion:** Overweight/obesity and hypertension were highly prevalent (>47% of adults >40 years are overweight or obese and >69% are hypertensive in the same age group) in our sample. Weight status was confirmed as a correlate of high blood pressure in our sample from Zanzibar, Tanzania. To early and effectively prevent related severe cardiovascular outcomes, screening strategies but also monitoring strategies are required for this population.

## Introduction

Hypertension is one of the leading causes of death worldwide (1, 2). But in contrast to other cardiovascular risk factors, the risk for hypertension is modifiable (3). While prevalence of hypertension in developed countries stabilizes around 35% or decreases, the prevalence rises in African countries. An increase of 89% from 2000 to 2025 is expected (4). By 2025 almost 75% of hypertensive patients will be found in developing countries (5). Today more than one third of the African adult population is hypertensive (2). And with 46% prevalence of hypertension in adults older than 25 years, Africa has the highest age-standardized hypertension prevalence worldwide (6). Due to few symptoms, hypertension is poorly detected and, even when detected, often untreated and barely controlled (6–8). Although the risk for hypertension increases with age, in sub-Sahara Africa, even young adults suffer from it (9, 10). Higher prevalence is found in lower socio-economic groups and follows demographic gradients from rural to urban areas (2, 3, 11). Overweight and obesity have been found to be modifiable risk factors for hypertension (12). Furthermore, salt intake is known as another important risk factor for development of hypertension. In African countries high concentrations of salt for food preparation and preservation has been established for a long time, due to poor possibilities of refrigeration or availability of gustatory ingredients (2).

Tanzania is one of Africa's low-income countries. Globalization as well as sedentary lifestyle has noticeable effects on health of the Tanzanian population (11). Like other developing countries, underweight and overweight/obesity and related comorbidities are present in the same population. This results in a rapidly increasing number of non-communicable diseases, such as hypertension and respective risk factors (13). Since obesity and salt intake are two reversible causes for high blood pressure (3, 14), we aimed to investigate associations between objectively measured anthropometric and biochemical markers such as urinary sodium and potassium excretion from morning spot urine and blood pressure in different age-groups in a study population from Zanzibar, Tanzania.

## Materials and Methods

### Study Design and Participants

**Figure 1:**
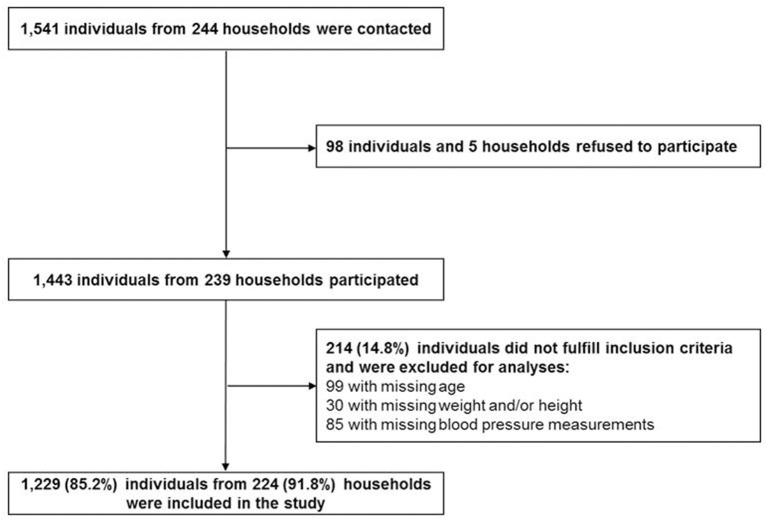
Flow diagram of participants from recruitment to analysis.

Data collection was conducted in a cross-sectional survey in 2013. Study participants were identified in 244 randomly selected households in Zanzibar, Tanzania. Power calculations, recruiting strategies and general measurement methods were described elsewhere (15). Briefly, the survey was carried out in 80 small administrative Wards (Shehias) in Unguja, one of the two main islands of Zanzibar. Survey sample and study sample are described in Figure 1. Overall, 1,541 individuals were contacted and 98 of them refused to participate. Of the 1,443 participants, 214 were excluded for this study due to missing information on age, weight, height and blood pressure measurements.

### Data Collection

Socio-demographic information on sex, age, and education level was collected through an interview-based questionnaire, which was completed for all participants above an age of 2 years. Information on young children were reported by their parents. Questionnaires were developed in English, translated into Swahili, and then back-translated to control for translation errors. All forms and questionnaires were administered and well-explained in Swahili by trained fieldworkers. Educational level of study participants was assessed using the International Standard Classification of Education (ISCED) (16). Based on this classification, we distinguished between low education (no or pre-primary education), primary education (primary school) and secondary education (secondary school and above).

Anthropometric measurements and collection of morning spot urine were carried out in fasting status following standardized procedures (17–19).

All anthropometric measurements and collection of biosamples were carried out considering standard operating procedures and study protocols. The measurement of proportion of body fat (% BF) and body weight was carried out barefoot using an electronic scale (TANITA BC-420 SMA, Germany) to the nearest 0.1 kg. Height of participants was measured to the nearest 0.1 cm using a stadiometer (Seca 213 stadiometer, UK) or a measuring board (Seca 417 measuring board, UK) for young children <2 years.

Body Mass Index (BMI) was calculated by dividing weight in kilograms by height squared in meters (kg/m^2^). For adults, overweight was defined as BMI ≥25 kg/m^2^; obesity was defined as BMI ≥30 kg/m^2^ according to the WHO (20). For children and adolescents up to an age of 19, BMI was transformed to age-and sex-specific z-score and percentiles using the WHO SAS macros “Anthro”^1^ and “AnthroPlus”^2^ in SAS 9.3 (SAS Institute Inc., Cary, North Carolina, USA). Categories for overweight (BMI between >75th and <95th percentile) and obesity (BMI > 95th percentile) were assigned according to the WHO centile curves (21). Waist circumference (WC) was measured at the midway point between the lower rib margin and the iliac crest in standing position and while wearing light clothing using an inelastic measuring tape (SECA 201) to the nearest 0.1 cm (22). % BF and WC were categorized into quartiles.

A digital automatic blood pressure monitor (Omron T3) was used to measure the systolic and diastolic blood pressure according to a standardized procedure (15). According to this, participants were asked to rest for at least 15 min before measurement. Measurements were taken on the naked right upper arm in sitting position with legs uncrossed. The middle of the blood pressure cuff has to be on heart level. Cuff length was determined according to the arm circumference, which was measured to the nearest 0.1 cm using an inelastic measuring tape (SECA 201). Two measurements were taken at an interval of 5 min plus a third measurement in case of >5% difference in blood pressure between the previous two readings. Mean of the two measurements with the smallest difference was taken into analyses. In case of similar differences between the three measurements, mean of all three measurements was taken. Classification was conducted with respect to participants' age. For children, blood pressure between the 90th and 95th percentile were classified as pre-hypertensive. Children with a blood pressure above the 95th percentile were classified as hypertensive (23). Adolescents and adults over 14 years with systolic blood pressure of ≥130 mmHg or diastolic blood pressure ≥80 mmHg were classified pre-hypertensive. Persons were classified as hypertensive, either if their systolic blood pressure was greater or equal 140 mmHg, their diastolic blood pressure reached a value of ≥90 mmHg (24) or regular use of anti-hypertensive medication was reported. As pre-hypertensive native African children have a high risk for developing hypertension in later life (25), pre-hypertensive as well as hypertensive children (2– ≤ 19 years) were classified as hypertensive for statistical analysis.

Urinary excretion of sodium, potassium and creatinine was measured in a 25 ml fasting status morning spot urine sample by ion-selective electrodes (ErbaLyteCaPlus) or picric acid assay (R&D creatinine kit). Twenty four hour urinary excretion of sodium and potassium was calculated from this single measurement using the Kawasaki formula (26). For using the Kawasaki formula, 24 h creatinine excretion was estimated according to another formula by Kawasaki et al. (27) accounting for age, sex, weight and height. Estimated excretion of sodium and potassium was used as surrogates for sodium and potassium intake.

### Statistical Analysis

Descriptive analyses were conducted to calculate sample characteristics regarding systolic and diastolic blood pressure, weight status and urinary excretion of sodium, potassium and creatinine as well as educational level and area of residence stratified by age-groups [young children (2– ≤ 9 years), children (>9– ≤ 19 years), young adults (>19– ≤ 40 years), adults (>40 years)] and sex. Statistics were provided in mean and standard deviation (SD) for continuous variables, and frequency (N) and proportions (%) for categorical variables.

Multilevel logistic regression was applied to calculate odds ratios (OR) and 95% confidence intervals (CI) in order to identify associations between anthropometric and biochemical markers with hypertension dichotomized as hypertensive and pre-hypertensive vs. normal blood pressure. Potential clustering within Shehias was considered in terms of a random intercept. With regard to a complete case analysis, item missing in variables included in the study, lead to differing sample size in each regression model. Quartiles of %BF and WC as well as BMI and 24 h sodium-to-potassium ratio were included as independent variables into each regression model. All models were adjusted for age, sex and area of residence and stratified by age-groups. Since education is highly correlated with age in children, adjustment for educational level for all participants ≤ 19 years was done using the highest educational level in their household. For adults, personal information on educational level was used.

We further adjusted for height in adults >19 years and height for age z-scores in children ≤ 19 years (23). Goodness of fit for all models was determined via the BIC. Statistical analyses were performed using SAS 9.3 (SAS Institute Inc., Cary, North Carolina, USA) and particularly mixed logistic regression models were conducted using the GLIMMIX procedure.

## Results

### Study Characteristics

In this study, a total of 1,229 participants, aged 2–95 years, fulfilled the inclusion criteria. Inclusion of study participants was described in Figure 1. The study sample consisted of 622 children and adolescents aged 2– ≤ 19 years with about 49% females and 607 adults aged 19+ years with 60% females (Table 1).

**Table 1:** Characteristics of the study population stratified by age and sex.

	**Young children (2–≤** **9 y.)** **(*****N*** **=** **261; 21.2%)**				**Children (>9–≤** **19 y.)** **(*****N*** **=** **361; 29.4%)**				**Young adults (>19–40 y.)** **(*****N*** **=** **327; 26.6%)**				**Adults (>40 years)** **(*****N*** **=** **280; 22.8%)**			
	**Male** **(*****N*** **=** **134; 51.3%)**		**Female** **(*****N*** **=** **127; 48.7%)**		**Male** **(*****N*** **=** **186; 51.5%)**		**Female** **(*****N*** **=** **175; 48.5%)**		**Male** **(*****N*** **=** **128; 39.1%)**		**Female** **(*****N*** **=** **199; 60.9%)**		**Male** **(*****N*** **=** **116; 41.4%)**		**Female** **(*****N*** **=** **164; 58.6%)**	
**Age [Mean (SD)]**	6.1	(1.9)	5.8	(1.9)	13.9	(2.8)	14.0	(2.7)	26.3	(6.3)	28.8	(6.6)	55.7	(10.1)	53.3	(9.8)
**Blood pressure [Mean (SD)]**																
SBP (in mm/Hg)	103.2	(10.9)	103.6	(12.6)	116.8	(13.5)	116.1	(11.8)	128.1	(13.5)	125.5	(17.0)	155.1	(27.2)	153.9	(32.4)
DBP (in mm/Hg)	66.6	(9.1)	67.6	(10.1)	70.5	(8.9)	72.9	(8.3)	78.1	(8.5)	79.5	(11.2)	90.8	(14.0)	89.6	(14.8)
**Hypertension [*****N*** (%)]^*^																
Yes	23	(17.2)	25	(19.7)	20	(10.8)	30	(17.1)	36	(28.1)	52	(26.1)	82	(70.7)	113	(68.9)
Prehypertension	21	(15.7)	15	(11.8)	31	(16.7)	20	(11.4)	25	(19.5)	35	(17.6)	16	(13.8)	14	(8.5)
**Weight status**^†^						
Severe thinness	71	(53.0)	52	(40.9)	42	(22.6)	25	(14.3)	7	(5.5)	14	(7.0)	2	(1.7)	5	(3.0)
Thinness	33	(24.6)	48	(37.8)	59	(31.7)	37	(21.1)	14	(10.9)	15	(7.5)	8	(6.9)	9	(5.5)
Normal weight	30	(22.4)	25	(19.7)	76	(40.9)	89	(50.9)	82	(64.1)	103	(51.8)	51	(44.0)	65	(39.6)
Overweight	0	(0.0)	2	(1.6)	6	(3.2)	16	(9.1)	24	(18.8)	41	(20.6)	40	(34.5)	37	(22.6)
Obesity	0	(0.0)	0	(0.0)	3	(1.6)	8	(4.6)	1	(0.8)	26	(13.1)	15	(12.9)	48	(29.3)
**Markers for weight status [Mean (SD)]**																
BMI (z-score)^‡^	−1.6	(1.0)	−1.4	(1.1)	−1.1	(1.2)	−0.5	(1.4)								
BMI									22.0	(3.5)	23.6	(5.0)	24.6	(4.5)	26.5	(6.9)
Body fat (in %)	14.9	(3.6)	14.6	(4.8)	10.5	(5.3)	20.4	(7.5)	15.6	(8.1)	27.9	(10.5)	21.7	(7.4)	34.3	(9.7)
Waist circum-ference (in cm)	50.4	(5.7)	49.5	(5.8)	62.7	(9.8)	64.9	(10.8)	76.4	(9.0)	78.7	(13.5)	87.1	(13.9)	88.6	(15.4)
**Urinary excretion [Mean (SD)]**																
Sodium (in mEq/day)^§^	181.7	(69.5)	139.7	(51.2)	191.6	(71.1)	168.5	(58.7)	196.4	(101.0)	172.5	(67.3)	182.2	(70.4)	178.3	(62.7)
Potassium (in mEq/day)^§^	36.4	(17.8)	27.9	(12.9)	40.1	(21.4)	33.4	(14.0)	38.7	(18.6)	36.9	(22.1)	38.8	(20.0)	36.5	(15.2)
24 h sodium- to-potassium ratio	5.4	(1.7)	5.6	(2.4)	5.3	(1.7)	5.4	(1.6)	5.3	(1.6)	5.1	(1.7)	5.1	(1.4)	5.2	(1.4)
Creatinine (in mg/ml)^||^	1.4	(1.0)	1.3	(0.9)	1.8	(1.3)	1.4	(1.0)	2.2	(1.3)	1.6	(1.1)	2.0	(1.2)	1.4	(1.1)
**Education [*****N*** **(%)]**^¶^																
None or primary	25	(18.7)	29	(22.8)	28	(15.1)	16	(9.1)	22	(17.3)	57	(29.5)	52	(46.4)	92	(56.8)
Secondary or higher	109	(81.3)	98	(77.2)	158	(85.0)	159	(90.9)	105	(82.7)	136	(70.5)	60	(53.6)	70	(43.2)
**Area of residence [*****N*** **(%)]**																
Rural	68	(50.8)	58	(45.7)	101	(54.3)	72	(41.1)	49	(38.3)	94	(47.2)	57	(49.1)	83	(50.6)
Urban	66	(49.2)	69	(54.3)	85	(45.7)	103	(58.9)	79	(61.7)	105	(52.8)	59	(50.9)	81	(49.4)

*SBP, Systolic blood pressure; DBP, diastolic blood pressure, BMI, body mass index*.

* *According to NIH (children) (23) and National Heart Foundation (adults) classification (24)*.

† *According to WHO classifications (21)*.

‡ *BMI z-score was calculated according to WHO (21)*.

§ *Estimated urinary excretion was determined from a fasting morning urine specimen on the basis of the Kawasaki formula (26)*.

|| *Measured creatinine from single morning spot specimen*.

¶ *According to UNESCO classification of educational levels (16). For children: highest education in household*.

Mean age for children and adolescents was 11 years and for adults 40 years in both sexes. Proportion of individuals from urban and rural residence was comparable in the sample with an exception in female children (>9– ≤ 19 years) and male young adults (>19– ≤ 40 y.), where about 60% of participants lived in urban areas.

**Figure 2:**
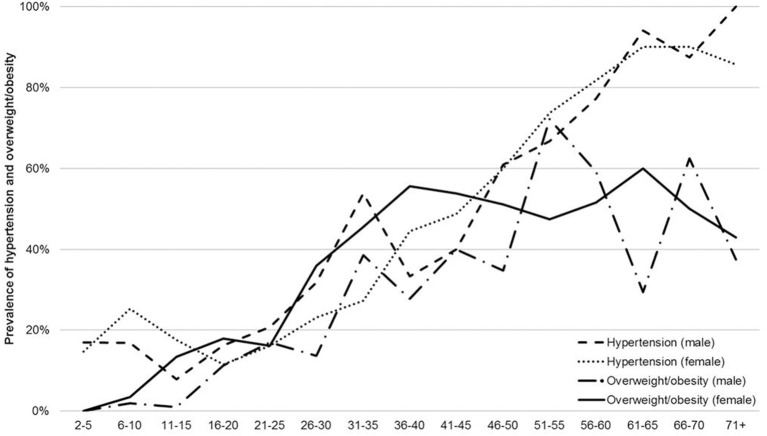
Prevalence of hypertension and of overweight/obesity.

Concerning participants' weight status, the highest percentage of participants per group changed from severe thinness in young children (2– ≤ 9 years) over thinness in children and adolescents (>9– ≤ 19 years) to normal weight or overweight in adults (>19 years). Systolic and diastolic blood pressure (Table 1, Supplementary Table 1) and the prevalence of hypertension and overweight/obesity increased with age (Figure 2, Supplementary Table 1).

### Associations Between Anthropometric and Biochemical Markers and Hypertension

**Figure 3:**
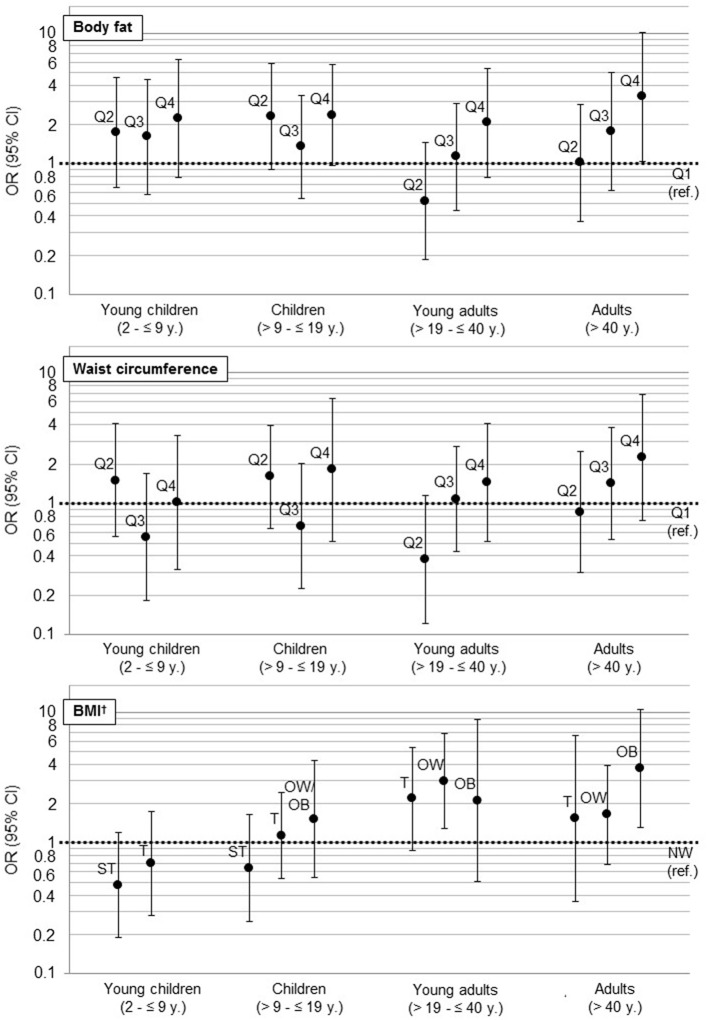
Association of anthropometric markers (WC, BF, BMI, ref: first Quartile) with hypertension as results from multilevel logistic regressions (hypertension: according to NIH (children) (23) and National Heart Foundation (adults) classification (24). For children: hypertension and prehypertension (25)). For a better visual comparison regarding the magnitudes of confidence intervals, we used a log scale. OR, Odds Ratio; CI, confidence interval; ref., reference; BMI, Body mass index; ST, severe thinness; T, thinness; NW, normal weight; OW, overweight; OB, obesity. †BMI z-score for children was calculated according to WHO (21).

Results of the mixed logistic regression models are shown in Figure 3 and Table 2. Young adults aged >19– ≤ 40 years, were more likely to be hypertensive or pre-hypertensive if they were overweight [OR = 3.0 (95% CI 1.3, 6.8)], but not obese compared to normal weight participants in the respective age-group. In adults >40 years, only obese [OR = 3.9 (95% CI 1.3, 11.1)] but not overweight participants were found to be more likely hypertensive compared to normal weight participants.

**Table 2:** Associated factors with hypertension^*^ detected in multilevel logistic regression models stratified by age.

		**OR (95% CI)**				**BIC**	**Between Shehia Variance (SE)**	
**Model**	***N***	**Marker for weight status**		**Sodium-to-potassium ratio**			
**Young Children (2–≤** **9 years)**								
Model 1(a): Bodyfat	170			1.0	(0.9, 1.2)	174.1	1.5	(0.5)
*Q1*		1	(Ref.)					
*Q2*		1.7	(0.7, 4.6)					
*Q3*		1.6	(0.6, 4.4)					
*Q4*		2.2	(0.8, 6.3)					
Model 1(b): Waist circumference	172			1.0	(0.9, 1.2)	170.3	1.5	(0.5)
*Q1*		1	(Ref.)					
*Q2*		1.5	(0.6, 4.1)					
*Q3*		0.6	(0.2, 1.7)					
*Q4*		1.0	(0.3, 3.4)					
Model 1(c):BMI^†^	172			1.0	(0.9, 1.2)	161.0	1.5	(0.4)
*Severe thinness*		0.5	(0.2, 1.2)					
*Thinness*		0.7	(0.3, 1.7)					
*Normal weight*		1	(Ref.)					
**Children (>9–≤** **19 years)**								
Model 2(a): Bodyfat	244			**0.7**	**(0.6, 0.9)**	311.3	1.1	(0.4)
*Q1*		1	(Ref.)					
*Q2*		2.3	(0.9, 5.9)					
*Q3*		1.3	(0.5, 3.3)					
*Q4*		2.4	(1.0, 5.8)					
Model 2(b): Waist circumference	244			**0.7**	**(0.6, 0.9)**	316.0	1.2	(0.4)
*Q1*		1	(Ref.)					
*Q2*		1.6	(0.7, 4.0)					
*Q3*		0.7	(0.2, 2.0)					
*Q4*		1.8	(0.5, 6.4)					
Model 2(c): BMI^†^	246			**0.7**	**(0.6, 0.9)**	320.4	1.2	(0.4)
*Severe thinness*		0.6	(0.2, 1.7)					
*Thinness*		1.1	(0.5, 2.4)					
*Normal weight*		1	(Ref.)					
*Overweight/obesity*		1.5	(0.5, 4.3)					
**Young Adults (>19**–**≤** **40 years)**								
Model 3(a): Bodyfat	198			1.0	(0.8, 1.2)	264.6	1.0	(0.4)
*Q1*		1	(Ref.)					
*Q2*		0.5	(0.2, 1.5)					
*Q3*		1.1	(0.4, 2.9)					
*Q4*		2.0	(0.8, 5.4)					
Model 3(b): Waist circumference	199			1.0	(0.8, 1.2)	266.7	1.1	(0.4)
*Q1*		1	(Ref.)					
*Q2*		0.4	(0.1, 1.2)					
*Q3*		1.1	(0.4, 2.7)					
*Q4*		1.4	(0.5, 4.0)					
Model 3(c): BMI	199			1.0	(0.8, 1.3)	265.6	1.1	(0.4)
*Thinness*		2.2	(0.9, 5.4)					
*Normal weight*		1	(Ref.)					
*Overweight*		**3.0**	**(1.3, 6.8)**					
*Obesity*		2.1	(0.5, 8.7)					
**Adults (>40 years)**								
Model 4(a): Bodyfat	186			1.3	(1.0, 1.7)	225.4	0.9	(0.5)
		1	(Ref.)					
		1.0	(0.4, 2.9)					
		1.8	(0.6, 5.0)					
		**3.3**	**(1.0, 10.1)**					
Model 4(b): Waist circumference	186			1.2	(0.9, 1.6)	227.6	1.0	(0.5)
		1	(Ref.)					
		0.9	(0.3, 2.5)					
		1.4	(0.5, 3.9)					
		2.2	(0.7, 6.8)					
Model 4(c): BMI	187			1.3	(1.0, 1.7)	224.7	0.9	(0.5)
*Thinness*		1.6	(0.4, 6.6)					
*Normal weight*		1	(Ref.)					
*Overweight*		1.6	(0.7, 4.0)					
*Obesity*		**3.7**	**(1.3, 10.5)**					

*All models were adjusted for age, sex, height [height for age for children ≤ 19 years (WHO)] and education (primary school and below was categorized as low and secondary school and above as high education (16); for children highest education was used as proxy). Random effects on Shehia level were considered*.

*OR, Odds Ratio; CI, confidence interval; BIC, Bayesian information criterion; SE, Standard Error, BMI, Body mass index*.

* *According to NIH (children) (23) and National Heart Foundation (adults) classification (24). For children: hypertension and prehypertension (25)*.

† *BMI z-score was calculated according to WHO (21). Bold numbers are statistically significant*.

When comparing the fourth to the first quartile of %BF, we also observed a significantly higher chance for hypertension in adults >40 years [OR = 3.4 (95% CI 1.1, 10.8)]. Furthermore, an increasing chance for hypertension was observed with increasing BMI as well as with increasing quartiles of %BF and WC in all age-groups, even though not significant.

We further observed a significant association between 24 h sodium-to-potassium ratio as marker for salt intake and hypertension in children aged >9– ≤ 19 years [OR = 0.7 (95% CI 0.6, 0.9), for all models (%BF, WC, BMI)]. Moreover, the direction of association between salt intake and hypertension changes with age. Children with higher salt intake, determined by urinary sodium-to-potassium ratio, were less likely to be hypertensive, whereas higher intake in adults over the age of 40 years was associated with hypertension. In adults, aged 19 to 40 years, salt intake showed no association with hypertension. Variance of random effects in mixed models varied from 0.9 in adults to 1.5 in young children, indicating a considerable amount of unexplained variance on the Shehia level (see Table 2).

## Discussion

This study was part of the SUTAS (Sustainable Use of Tropical Aquatic Systems) survey and was conducted in order to identify anthropometric and biochemical associations with hypertension and pre-hypertension in Unguja Island, Zanzibar, Tanzania. This was the first epidemiological study in Unguja Island, Zanzibar, investigating associations between markers of overweight/obesity and salt intake and hypertension using standardized methods in a randomized sample of members of a household. The most recent surveillance report from the Zanzibar observed systolic and diastolic blood pressure rates and hypertension proportions that were for men and women (age-groups 25–44 years and 45–64 years) lower compared to the respective age-groups of the present study (28). Recently, there have been studies on hypertension in Tanzania (7, 29, 30), but most of them focus on the adult population of the mainland (7, 30). To our knowledge this is the first study providing information on associations between anthropometric and biochemical markers and hypertension not only in adult populations but also for Zanzibari children from an age of 2 years onwards.

In our sample, prevalence of hypertension increased with age, and thus supports the findings of other studies in Tanzania (7, 29). Overall, about 16% of children (2– ≤ 19 years) in the study population suffered from hypertension. A recent meta-analysis on prevalence of elevated blood pressure in children and adolescents in Africa reported on a prevalence range from 0.2 to 24.8% (31). Pooling all studies, they found a pooled prevalence of 5.5% for blood pressure >95^th^ percentile. However, pooled prevalence in Eastern Africa was found to be higher (9.0%). In addition, our results are comparable to the reported hypertension prevalence (8.4% up to 24.4%) in a South African study investigating children between 5 and 18 years (25). Additionally, 14% of children (2– ≤ 19 years) in our study sample were pre-hypertensive, which corresponds to findings from the respective South African sample and the recent meta-analysis (25, 31). In adults (>19 years), prevalence of hypertension was 47% and considering also the prevalence of pre-hypertension (15%), the hypertension prevalence in our sample was higher compared to the hypertension prevalence in the whole of Tanzania (39%), reported by WHO in 2011 (32). However, considering only the age group of adults (>40 years) we found a hypertension prevalence of about 70%, which is similar to the results of a comparable study in adults older than 50 years in South Africa (33).

BF% and WC are good indicators for children's weight status (34), but hypertension in children is not only determined by weight but also by height (23). Considering BF%, WC and BMI as different markers for weight status, logistic regression models including BMI showed the best goodness of fit in young children (2– ≤ 9 years). This indicates that BMI, including not only weight but also height, seems to be a better predictor for hypertension than other markers of weight status in young children. Participants with higher BMI seemed to be more likely to be hypertensive in all age-groups. A lower BMI was associated with an increased chance for hypertension as well. Only severe thinness in children <19 years was found to be preventive regarding high blood pressure. Whereas, models for WC and %BF seems to correlate for the adult population (Figure 3), they were less precise in young (2– ≤ 9 years) and in older children (>9– ≤ 19 years), due to a small number of hypertensive participants and a high prevalence of underweight in these age-groups. Furthermore, WC and %BF as indicator for weight status did not correlate with weight status compared to BMI in our sample: children with (severe) thinness, which were possibly suffering from severe acute malnutrition, such as diseases like Marasmus or Kwashiorkor and might have mislead the classification for markers of weight status due to an altered body composition (35). Additionally, the small sample size may have prevented us from detecting an association between weight status and hypertension in these age-groups.

Since interventions on salt reduction in Sub-Sahara Africa resulted in improved outcome measures of blood pressure and urinary sodium excretion (14), we furthermore expected an association between sodium-to-potassium ratio (as substitute for salt intake) and hypertension in our sample. Previous studies reported that high intake of salt (36–39), especially sodium, was associated with hypertension (40), whereas potassium seemed to act as a preventive factor (41). But contrary to previous findings, we observed a significant decreased hypertension chance associated with a high sodium-to-potassium ratio (higher sodium than potassium excretion) in the age-group >9– ≤ 19 years. In addition, we observed that a high sodium-to-potassium ratio reduce chance of hypertension (OR <1) in young children (2– ≤ 9 years) and children (>9– ≤ 19 years) as mentioned, but in young adults (>19– ≤ 40 years) no association was found, while a high sodium-to-potassium ratio was found to be apparent associated (OR > 1) with hypertension in adults (>40 years). However, these results should be interpreted carefully. A lower excretion of potassium than sodium in children does not necessarily indicate lower potassium intakes. Potassium is needed especially in somatic growth (42) and can therefore affect the sodium-to-potassium ratio in urine. However, we could not provide a reasonable justification for the observed positive association between lower potassium excretion and a decreased chance of hypertension. Further influencing factors like genetic predispositions (43–47) and early life origins (48, 49) should be taken into consideration.

We observed a considerable amount of unexplained variance on the Shehia level, which was more pronounced in children than in adults (see Table 2). Urban and rural areas often differ in topography, distances to daily supplies, access to food and medical services as well as sources of income. Due to the random distribution of Shehias throughout Zanzibar, such area and Shehia level confounders were not measured.

Regarding strengths and limitations, this survey is one of the first ones including an exhaustive study protocol on Zanzibar, Tanzania. It covers anthropometric measurements and biochemical markers of all age-groups in different regions of Unguja Island. Unfortunately, proportion of children <5 years of age per household was lower than expected (50) which may have attenuated the effects for the age-group 2 to ≤ 9 years (mean age 11 years) due to a smaller sample size. The full sample included individuals from households and Shehias that were both randomly selected. We thus assume that the study sample characterizes the Zanzibari population. Additionally, using the Kawasaki formula we were able to consider creatinine concentration of the urine sample provided, which is a clear strengths of our study. We also investigated a possible non-linear association between the sodium-to-potassium ratio and blood pressure in the subsample of salt intake (results not shown). However, in sensitivity analyses cubic regression splines fitted similarly compared to a simple linear univariate regression; thus, we kept the originally conducted linear regression models.

Since the study was performed in a cross-sectional design, it is not possible to attribute the observed significant associations as causal. Therefore, we suggest confirming the reported findings using longitudinal data of a comparable study population. Furthermore, other possible determinants like physical activity could not be taken into account. Although physical activity was measured in this study by uniaxial accelerometer, we did not use the data in our analysis. Data were available only for a small subsample of children and adolescents, aged between 3 and 16 years, which were randomly selected from 20 Shehias for these measurements. Additionally, there is always a degree of uncertainty with regard to fasting status of participants for at least 12 h prior to collection of biological samples. However, self-collection of urine potentially affected our data.

Assessing plausible salt intakes is difficult. We therefore decided to use urinary excretion of sodium and potassium as surrogates. Even if previous research indicates that urinary excretion could be used as surrogate for salt intake (51), a difference between intake and excretion cannot be ruled out completely. To our knowledge, there are no existing formulas to calculate 24 h salt excretions from single morning spot urine that were validated in a sub-Sahara African population for adults as well as for children. We conducted sensitivity analyses using the formulas from Kawasaki et al. (26), from Tanaka et al. (52) and from INTERSALT (53). We did not observe remarkable differences in results when applying different formulas on our study data (data not shown). We decided to use the Kawasaki formula (26), since a consideration of both sodium and potassium is possible using this method.

The interpretation of the association between hypertension and salt excretion from a single measurement had to be considered carefully, since previous studies showed that four to seven urine specimens are needed to estimate sodium-to-potassium ratio in hypertensive individuals (54).

## Conclusion

Despite the limitations this study to our knowledge is the first to report such associations in a randomly selected population using standardized assessment of anthropometrical and laboratory measurements in Zanzibar.

High blood pressure among Zanzibaris is positively associated with BMI and body composition in >19–95 year olds and is negatively associated with salt intake in >9– ≤ 19 year olds. Still, there is a tendency that salt intake increases hypertension chance in the age-group 40 years and above. Promotion strategies to reduce hypertension prevalence among Zanzibaris should consider healthy weight maintenance and strategies that facilitate appropriate salt intakes for the population.

For the future, a reduction of the high hypertension prevalence is of high public health interest. There is a need for effective interventions to prevent long-term effects of hypertension and for primary prevention strategies to tackle the rising prevalence using local multidisciplinary approaches considering local lifestyles and eating habits in the local language, Swahili. Continuation of health surveillance initiatives will help to monitor health prevention activities in future. Furthermore, practicing physicians and health professionals should proactively advice and teach patients self-care skills in diet for weight maintenance or weight loss to prevent development of cardiovascular diseases.

## Ethics Statement

We certify that all applicable institutional and governmental regulations concerning the ethical use of human volunteers were followed during this research. The study was performed according to the Helsinki Declaration and the study protocol was evaluated and approved by the Ethics Committees of the University of Bremen and of the Zanzibar Ministry of Health and the Zanzibar Medical Research and Ethics Committee. Study participants did not undergo any procedures unless both children and their parents had given consent for examinations, collection of samples, subsequent analysis and storage of personal data and collected samples. Study subjects could consent to single components of the study while abstaining from others. All participants agreed and signed that the data collected during the survey can be stored and used for future analysis.

## Author Contributions

AH was a principal investigator and had the idea of the analysis. LB wrote the paper and had primary responsibility for final content. CB provided advise for the data analysis. MN involved in the development of the instruments and was responsible for the survey and data management. LB, CB, MN, AH, SK, and MS were responsible for critical revisions and final approval of the manuscript.

### Conflict of Interest

The authors declare that the research was conducted in the absence of any commercial or financial relationships that could be construed as a potential conflict of interest.

## Footnotes

^1^https://www.who.int/childgrowth/software/en/

^2^https://www.who.int/growthref/tools/en/

## Abbreviations

ISCED: International Standard Classification of Education
% BF: proportion of body fat
BMI: Body Mass Index
NIH: National Institutes of Health
WHO: World Health Organization
WC: waist circumference
SUTAS: Sustainable Use of Tropical Aquatic Systems.
