# Supplementary material for: Anthropometric and Biochemical Predictors for Hypertension in a Cross-Sectional Study in Zanzibar, Tanzania
Source: Front Public Health. 2019 Nov 21;7:338. doi: 10.3389/fpubh.2019.00338 (PMC6881248; doi:10.3389/fpubh.2019.00338)
Supplement: Supplementary file 1 [file Table_1.DOCX]

Supplementary Table 1: Prevalence of hypertension and of overweight/obesity

| **Age (years**) | **Male** | | | | | **Female** | | | | |
| --- | --- | --- | --- | --- | --- | --- | --- | --- | --- | --- |
|  | **N** | **Hypertensives (N (%))** | | **Overweigt/obese (N(%))** | | **N** | **Hypertensives (N (%))** | | **Overweigt/obese (N(%))** | |
| 2-5 | 53 | 9 | (17.0 %) | 0 | (0.0 %) | 61 | 9 | (14.8 %) | 0 | (0.0 %) |
| 6-10 | 107 | 18 | (16.8 %) | 2 | (1.9 %) | 87 | 22 | (25.3 %) | 3 | (3.4 %) |
| 11-15 | 102 | 8 | (7.8 %) | 1 | (1.0 %) | 97 | 17 | (17.5 %) | 13 | (13.4 %) |
| 16-20 | 80 | 13 | (16.3 %) | 9 | (11.3 %) | 78 | 9 | (11.5 %) | 14 | (17.9 %) |
| 21-25 | 53 | 11 | (20.8 %) | 9 | (17.0 %) | 62 | 10 | (16.1 %) | 10 | (16.1 %) |
| 26-30 | 22 | 7 | (31.8 %) | 3 | (13.6 %) | 39 | 9 | (23.1 %) | 14 | (35.9 %) |
| 31-35 | 13 | 7 | (53.8 %) | 5 | (38.5 %) | 33 | 9 | (27.3 %) | 15 | (45.5 %) |
| 36-40 | 18 | 6 | (33.3 %) | 5 | (27.8 %) | 45 | 20 | (44.4 %) | 25 | (55.6 %) |
| 41-45 | 20 | 8 | (40.0 %) | 8 | (40.0 %) | 39 | 19 | (48.7 %) | 21 | (53.8 %) |
| 46-50 | 23 | 14 | (60.9 %) | 8 | (34.8 %) | 45 | 27 | (60.0 %) | 23 | (51.1 %) |
| 51-55 | 18 | 12 | (66.7 %) | 13 | (72.2 %) | 19 | 14 | (73.7 %) | 9 | (47.4 %) |
| 56-60 | 22 | 17 | (77.3 %) | 13 | (59.1 %) | 33 | 27 | (81.8 %) | 17 | (51.5 %) |
| 61-65 | 17 | 16 | (94.1 %) | 5 | (29.4 %) | 10 | 9 | (90.0 %) | 6 | (60.0 %) |
| 66-70 | 8 | 7 | (87.5 %) | 5 | (62.5 %) | 10 | 9 | (90.0 %) | 5 | (50.0 %) |
| 71+ | 8 | 8 | (100.0 %) | 3 | (37.5 %) | 7 | 6 | (85.7 %) | 3 | (42.9 %) |
